# Egg-Independent Influenza Vaccines and Vaccine Candidates

**DOI:** 10.3390/vaccines5030018

**Published:** 2017-07-18

**Authors:** Ilaria Manini, Claudia Maria Trombetta, Giacomo Lazzeri, Teresa Pozzi, Stefania Rossi, Emanuele Montomoli

**Affiliations:** 1 Department of Molecular and Developmental Medicine, University of Siena, Via Aldo Moro 2, 53100 Siena, Italy; trombetta@unisi.it (C.M.T.); giacomo.lazzeri@unisi.it (G.L.); teresa.pozzi@unisi.it (T.P.); stefania.rossi@unisi.it (S.R.); emanuele.montomoli@unisi.it (E.M.); 2 VisMederi S.r.l., Strada del Petriccio e Belriguardo 35, 53100 Siena, Italy

**Keywords:** cell-culture, vaccination, synthetic influenza vaccine

## Abstract

Vaccination remains the principal way to control seasonal infections and is the most effective method of reducing influenza-associated morbidity and mortality. Since the 1940s, the main method of producing influenza vaccines has been an egg-based production process. However, in the event of a pandemic, this method has a significant limitation, as the time lag from strain isolation to final dose formulation and validation is six months. Indeed, production in eggs is a relatively slow process and production yields are both unpredictable and highly variable from strain to strain. In particular, if the next influenza pandemic were to arise from an avian influenza virus, and thus reduce the egg-laying hen population, there would be a shortage of embryonated eggs available for vaccine manufacturing. Although the production of egg-derived vaccines will continue, new technological developments have generated a cell-culture-based influenza vaccine and other more recent platforms, such as synthetic influenza vaccines.

## 1. Introduction

Influenza occurs globally, its annual attack rate being estimated at 20–30% in children and 5–10% in adults. Illness can result in hospitalization and death, mainly among high-risk groups. Worldwide, annual epidemics are estimated to cause around 3 to 5 million cases of severe illness and 250,000 to 500,000 deaths [1]. Influenza A viruses are responsible for annual epidemics and sporadic pandemics. In one study, 99% of hospitalized cases were caused by influenza A and only 1% by influenza B [2]. Influenza epidemics occur in the northern hemisphere from October to March and in the southern hemisphere from April to September. The Global Influenza Surveillance and Response System (GISRS) has been performing influenza virological surveillance since 1952. Since its inception, the network has developed and now consists of over 143 National Influenza Centers (NICs) in 113 countries, and 6 Word Health Organization (WHO) Collaborating Centers (CCs) [3]. Together, these laboratories process around 500,000 respiratory specimens per year in order to monitor influenza activity around the world. The NICs send approximately 8000 of the viruses isolated to the CCs for antigenic and genetic characterization, in order to support the selection of strains for the composition of the seasonal influenza vaccine.

The production of an optimal influenza vaccine requires constant global influenza monitoring for the emergence and circulation of new viruses other than those circulating in the previous season [4,5]. Most seasonal flu vaccines are hen egg-derived (Table 1) in the European Union/European Economic Area (EU/EEA) [6]. The influenza virus is grown in the allantoic cavity of embryonated hen eggs; the virus is then harvested, inactivated, purified and processed [7]. With current manufacturing capacity, 413 million doses of a trivalent influenza vaccine are produced every year for the world population [8], and in some EU/EEA countries quadrivalent inactivated influenza vaccines are expected to substitute the trivalent formulation over time.

Also in the US, the ordinary way that flu vaccines are made is using an egg-based manufacturing process for trivalent and quadrivalent inactivated and live attenuated influenza vaccine (Table 2) [9]. For the 2016–2017 season from 157 to 168 million doses of injectable influenza vaccine have been distributed in US [10].

**Figure 1:**
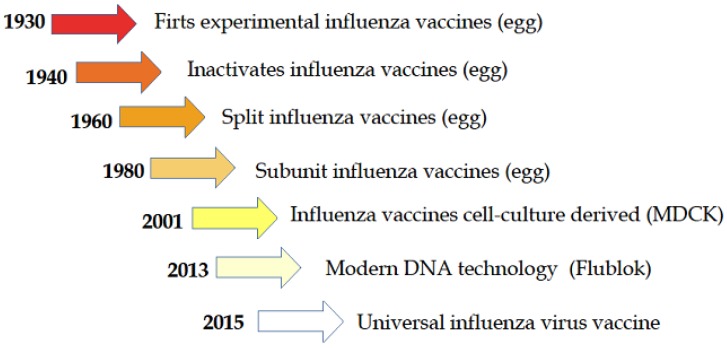
Historical path of the development of influenza vaccine [11].

Today, three parallel approaches for producing influenza vaccines are attracting the interest of many vaccine manufacturing companies; the first and oldest is the conventional egg-derived influenza vaccine, the second is a cell culture-derived influenza vaccine, and the third and most recent technology is the production of synthetic vaccines (Figure 1).

## 2. Cell Culture-Derived Vaccines and Vaccine Candidates

In 1995, the WHO recommended developing an alternative influenza virus cultivation system and investigating mammalian cell culture lines [12]. Three cell lines were commercially proposed for cell culture-derived influenza vaccines: Madin Darby canine kidney cells (MDCK), Vero cells (Kidney epithelial cells from an African green monkey), used for more than twenty years for polio vaccine production [13], and PER.C6, a human retina-derived cell line.

MDCK cells and Vero cells were shown to be especially promising cell-line candidates because, at that time, influenza vaccines were produced only in embryonated hen eggs, and therefore, required large numbers of high-quality fertile hen eggs, which could only be obtained by careful advance planning. In the case of a pandemic strain, sufficient quantities of hen eggs may not be readily available to produce specific vaccines because the timeline from strain identification to vaccine is about four-six months [8], and between one to two eggs are usually required to produce one influenza vaccine dose. Growing the influenza virus in the egg can lead to adaptations in which different phenotypes from the wild virus circulating in humans are selected, thus egg based vaccines could possibly be contraindicated for people with allergies to egg components [14]. The MDCK cell line was established from the kidney of a healthy cocker spaniel by SH Madin and NB Darby at the University of California, Berkeley, in 1958. The MDCK 33016 suspension cell line has been adapted to grow under serum-free conditions [15], and MDCK cells have been used to isolate a wide variety of human influenza viruses [16]. The production of cell culture-derived influenza vaccines has many advantages. Firstly, the long lead times required by egg-based production systems are eliminated, and there is a more controlled production process involving closed-system bioreactors. Moreover, viruses propagated in mammalian-derived tissue culture remain antigenically unchanged, unlike the case of incubation in embryonated eggs, because this host system induces a selection of variants that grow well [17]. On 1 June 2007, Optaflu, a MDCK cell culture-derived seasonal influenza vaccine was manufactured by Novartis Vaccines and approved by the European Medicines Agency (EMA) for intramuscular use in the EU [18]. It took five years before Novartis Vaccines could market Optaflu. Indeed, the first clinical trials had begun in Germany in 2002 with a Phase I study involving 40 volunteers aged 18–40 years; this was followed in 2003 by a Phase II study involving 200 volunteers aged 18–60 years, and by Phase III, studies in the subsequent years up to post-marketing studies [19,20].

In November 2012, the Food and Drug Administration (FDA) approved the first cell culture-derived influenza vaccine, Flucelvax. As the original brand name of this product, Optaflu, was deemed unacceptable by the Center for Biologics Evaluation and Research (CBER) in the US, the name “Flucelvax” was proposed and accepted [21].

German regulatory authorities approved Celtura (Novartis), an MF59-adjuvanted, MDCK-CCIV A/H1N1, cell culture-derived pandemic vaccine, in November 2009. A phase I, randomized study of Celtura from July to September 2009 showed an antibody response plausibly associated with protection after the administration of a single dose [22]. A post-licensure vaccine surveillance study conducted in 2012 confirmed the good safety profile of Celtura (Table 3) [23].

By 1998, Baxter Vaccines had already developed a Vero cell-based process to produce a new vaccine derived from cell culture [7]. This new line of research, a Vero cell-derived pandemic vaccine based on a wild-type A/California/2009 H1N1 strain, allowed Baxter to license in Europe. European regulatory authorities approved this vaccine, Celvapan, in October 2009 [24]. Preflucel, also manufactured by Baxter, is a seasonal influenza vaccine based on a Vero cell-line platform to produce three inactivated influenza viruses, including the A/H1N1 pandemic strain. During the 2008–2009 influenza season, a Phase III clinical study of Preflucel was conducted in the US. This demonstrated that a Vero-derived vaccine was safe and well tolerated in both youths and adults [25,26]. However, on 20 October 2011, the EMA was informed by the Austrian Medicines Regulatory Agency that Baxter had recalled large batches from the EU market owing to increasing suspicion of side effects; as a precautionary measure, the EMA then recalled all batches from European markets (Table 3) [27]. PER.C6 cell lines have been shown to meet both EU and US regulatory requirements for the production of influenza vaccines. Having obtained a license to use PER.C6 for influenza vaccine production, Sanofi Pasteur started a Phase I clinical trial of their H7N1 vaccine in 2009 (Table 4). This was the first study conducted on a cell-based H7 pandemic virus vaccine candidate, and, although the vaccine was well tolerated, the results showed poor immunogenicity and humoral immune responses; thus, the vaccine did not meet the criteria for vaccine approval of the Committee for Medicinal Products for Human use (CHMP) [28]. To date, no flu vaccines derived from PER.C6 have been approved for use in humans (Table 5). 

## 3. New Technologies for New Influenza Vaccines

Cell production of the influenza vaccine has some limitations; several companies currently use the MDCK and the Vero-adherent cell line although scale-up is the biggest challenge. Other non-adherent cell lines used for the production of influenza vaccines such as PER.C6 have yielded unsuccessful results in their first clinical studies concerning the immune response of the vaccine. The live Influenza virus needs inactivation and/or attenuation throughout different steps of biocontainment, and consequently produces a low yield of the final product, which represents a major limitation for this technology [29].

To attempt to overcome the limitations of cell culture vaccine production, such as the need to propagate the virus in cells, or possible cell culture contamination, new influenza vaccines have been produced by means of a baculovirus expression vector system (BEVS) instead of cell-line culture. In this process, the Hemagglutinin (HA) gene sequence is inserted into an insect cell containing a baculovirus. The insect cells are grown to modest density in an animal-free suspension culture serum [30]. In the vaccine composition, the hemagglutinin is 45 µg for each viral strain than 15 µg of other seasonal vaccines [31]. On 16 January 2013, the FDA approved Flublok, the first licensed vaccine using an insect virus expression system, produced by Protein Sciences Corporation. Other influenza vaccines are being developed by means of new technologies, especially pandemic influenza vaccines. A Phase I study in healthy adults has demonstrated the safety and immunogenicity of a plant-produced recombinant hemagglutinin influenza vaccine derived from the A/H1N1 pandemic strain. Specifically, infecting the tobacco plant *Nicotiana benthamiana* with a hybrid vector containing the specific viral HA allows large-scale production of a recombinant hemagglutinin protein [32]. The first study conducted in humans assessed the immunogenicity of HAC1, a plant-produced recombinant HA influenza vaccine [33]. Several viruses are used as recombinant vector vaccines; for example, a modified vaccinia virus Ankara (MVA) has been used as a vaccine vehicle of the foreign HA gene of the H5N1 influenza virus [34]. A recent phase I-II study involving 80 volunteers aged 18–29 years has demonstrated the tolerability and immunogenicity of this vaccine. However, its immunogenicity has not yet been compared with that of conventional H5N1 inactivated vaccines [35]. An alternative formulation for influenza vaccines involves the use of a DNA-encoding influenza virus nucleoprotein, which is administered through intramuscular injection. DNA nucleoprotein injection in mice induces protection against homologous and heterologous virus strains [36]. Another strategy is the use of a chimeric HA (cHA) for to formulate a universal influenza vaccine, a method completely different from those mentioned so far. Since the immune system always recognizes the same domain of the HA stalk, even if the head of the various influenza viruses is different there would be a booster response to the vaccine without needing to repeat the vaccination before each influenza season. With this innovative technique, all kinds of influenza vaccines could be produced, like inactivated whole vaccines, split, live attenuated vaccines, recombinant vaccine, and DNA vaccines.

It has also been observed in the laboratory that when the immunodominant globular domain is removed the antibody response to the HA stem increases [37,38].

## 4. Conclusions

Although several companies continue to produce subunit egg-derived vaccines, new manufacturing platforms are being developed for new influenza vaccines.

The first influenza vaccine produced in eggs was licensed in 1945. Egg-derived and cell-derived influenza vaccines will likely be produced and marketed in parallel in the next decade. The development of faster and more innovative technologies will shorten waiting times in comparison with egg-based vaccine production.

The production of vaccines by means of cell culture technologies has several advantages: Cell culture manufacturing is cleaner and faster, which is especially important in the case of a pandemic; the phenomenon of virus non-adaptation is avoided, and, lastly, the growing cell is controlled in defined culture media and validated cell banks in accordance with Good Manufacturing Practice (GMP), in contrast with the less strict requirements applied to egg-based vaccine production.

So far, the two most successful cell lines have been the MDCK cell line and the Vero cell line, the use of which has enabled seasonal and pandemic influenza vaccines to be manufactured and marketed in Europe and the US.

Vaccine production in cell cultures has both advantages and a few disadvantages. A clear advantage is that any adventitious infective agents in cell cultures can be detected and removed, while a major disadvantage is that pre-existing facilities need to be either adapted or completely rebuilt [39]. Every step of the influenza vaccine manufacturing process is steadily monitored because there are many possibilities for contaminates : the presence of adventitious viruses in the original clinical isolate, oncogenic viruses in cell cultures, production operators or raw materials [40].

New technological discoveries have led to state-of-the-art latest-generation influenza vaccines, in which the vaccine is created without inactivating the influenza virus or using a subunit surface; thus, many of the problems inherent in egg-based and cell-based production can be avoided. The great advantage of synthetic vaccines is that the production process does not start with the whole virus, from which the proteins of interest then have to be extracted; rather, the nucleotide sequence of a glycoprotein, such as hemagglutinin HA, is artificially synthesized and inserted into a productive context that is completely independent from the virus [41,42]. DNA vaccines may be future candidates for human vaccination, and pre-constituted libraries of vaccine strains may be utilized in the event of epidemics and, especially, pandemics [43].

## Figures and Tables

**Table 1:** Available seasonal influenza vaccines in the EU/EEA (2015/2016 season).

Manufacturer	Name of Product	Vaccine Type	Adjuvant	Produced in	Age Recommended
Abbot healthcare	Trivalent: Influvac Imuvac	Inactivated	None	Egg	From 6 months
AstraZeneca	Quadrivalent: Fluenz Tetra (Flumist quadrivalent) Trivalent: Fluarix Alpharix Influsplit	Live attenuated	None	Egg	From 24 months to 17 years Trivalent: From 6 months
GlaxoSmithKline	Quadrivalent: Fluarix Tetra Alpharix Tetra Influsplit Tetra	Inactivated/split	None	Egg	From 3 years
Novartis	Trivalent: Agrippal Fluvirin Fluad	Inactivated/subunit	None None Squalene (MF59)	Egg	From 6 years From 4 years From 65 years
Pfizer/CSl Australia	Trivalent: Afluria Enzira	Inactivated	None	Egg	From 5 years
Sanofi Pasteur	Trivalent: Vaxigrip Intanza 9 µg Intanza 15µg	Inactivated	None	Egg	From 6 months From 18-59 years From 60 years

**Table 2:** Available seasonal influenza vaccines in the US (2016/2017 season).

Manufacturer	Name of Product	Vaccine Type	Adjuvant	Produced in	Age Recommended
GlaxoSmithKline	Quadrivalent: Fluarix	Inactivated/split	None	Egg	From 3 years
ID Biomedical Corp. of Quebec (distributed by GlaxoSmithKline)	Quadrivalent: FluLaval	Inactivated/split	None	Egg	From 3 years
Sanofi Pasteur	Quadrivalent: Fluzone Quadrivalent: Fluzone Intradermal Trivalent: Fluzone high dose	Inactivated/split Inactivated/split/intradermal Inactivated/split	None	Egg	From 6 through 35 months From 36 through 8 years From 9 years From 18 through 64 years From 65 years
Seqirus	Trivalent: Afluria Fluvirin Fluad	Inactivated/subunit Inactivated/subunit/adjuvanted	None Yes	Egg	From 5 through 8 years From 4 years From 65 years
MedImmune	Quadrivalent: FluMist	Live/attenuated	None	Egg	From 2 through 49 years

**Table 3:** Summary information on published Clinical Trial of Madin Darby canine kidney (MDCK) cell culture-derived influenza vaccine, Novartis.

Vaccine	Year of Study Data	Phase	Trial Participants	Results: Immunogenicity
Optaflu	2002	I and II, randomized, observer-blind, controlled, single-center	240 healthy adults aged 18–40 years and 120 elderly aged > 61 years	Both Optaflu and control vaccine met all CHMP criteria in hemagglutination inhibition assay
Optaflu	2004–2005	III, randomized, observer-blind, controlled, multicenter	1300 healthy adults aged 18–40 years and 1354 elderly aged > 61 years	Both Optaflu and control vaccine met all CHMP criteria.
Optaflu	2005–2006	III, randomized, observer-blind, controlled, multicenter, lot-to-lot variability	1200 healthy adults aged 18–40 years	Both Optaflu lots and control vaccine met all three CHMP criteria
Celtura	2009	I, single center	176 adults	Satisfactory immune responses

**Table 4:** Summary information on published Clinical Trial of Vero cell culture-derived influenza vaccine, Baxter.

Vaccine	Year of Study Data	Phase	Trial Participants	Results: Immunogenicity
Celvapan	2009	I and II, prospective, randomized, open label, multicentre	200 adults and 200 elderly	Vaccine met criteria for immunogenicity responses
Preflucel	2008-2009	III, multicenter, randomized, double-blind,	7250 adults, 3210 older	Vaccine met criteria for immunogenicity responses

**Table 5:** Summary information on published Clinical Trial of PER.C6 cell culture-derived influenza vaccine, Sanofi Pasteur.

Vaccine	Year of Study Data	Phase	Trial Participants	Results: Immunogenicity
n/a	2009	I, randomized, open label	60 adults	Poor immunogenicity and humoral immune responses
